# Supplementary material for: Are carbon emissions trading and green financial instruments synergistic? -Comprehensive quantitative research based on content analysis
Source: PLoS One. 2024 Mar 7;19(3):e0298601. doi: 10.1371/journal.pone.0298601 (PMC10919601; doi:10.1371/journal.pone.0298601)
Supplement: S1 Data — (ZIP) [file pone.0298601.s001.zip › Data/地级市Z_F工作报告.zip!/─┌├╔╣┼/║⌠┬╫▒┤╢√╩╨/└┤╫╘╣1⁄2╓┌║┼í╛┬φ┐╦╩2╛▌═°í┐_2012.docx]

各位代表：

现在，我代表呼伦贝尔市人民政府向大会报告工作，请予审议，并请市政协委员和列席会议的同志提出意见。

一、２０１１年工作回顾

２０１１年，面对国际经济形势复杂、国内宏观调控政策趋紧、节能减排压力增大等困难，在自治区党委、政府和市委的领导下，坚定实施市第三次党代会提出的“美丽发展、科学崛起、共享繁荣”发展战略，继续推进“五区”建设，保持了经济快速发展、民生持续改善、社会和谐稳定，实现了“十二五”时期的良好开局。

（一）经济保持了强劲发展势头。地区生产总值和地方财政总收入实现历史性突破，分别跨过千亿和百亿大关，达到１１４５．３亿元和１１４．４亿元，分别增长２２．９％和２２．７％。固定资产投资完成７７４．３亿元，增长２７．３％。“双百亿”工程引领工业高位运行，规模以上工业总产值达到１０４０．７亿元，增长４４．３％。农牧业再获丰收，粮食总产量达１０５亿斤，增长４．９％；牧业年度牲畜存栏１６５２万头只，畜群结构进一步优化；农牧业产业化发展步伐加快，销售收入百万元以上加工企业达到１５１户，实现销售收入２１１．１亿元，增长３１．５％。旅游接待游客１１６５万人次，增长１９％；旅游业总收入达到１７３亿元，增长２１％。服务业发展步伐加快，社会消费品零售总额完成３４３．９亿元，增长１８．９％。对外开放取得新进展，满洲里国家重点开发开放试验区顺利推进，中蒙跨境合作区取得积极进展，进出口总额完成２５．７亿美元，增长１０．３％；引进到位资金５９１亿元，增长１８．７％；实际利用外资９７２９万美元，增长１５％。全面完成自治区下达的节能减排目标任务。

（二）经济结构调整初见成效。以投资和项目为引领，加大经济结构调整力度。工业投资４２６．５亿元，增长１７．１％；农牧业产业化投资４６．０６亿元，增长５４％；城市建设投资１９２．２亿元，增长５１％；社会事业投资７０．８８亿元，增长３６．７％。投资结构调整带动经济结构调整，三次产业结构由１９．６４２．１３８．３演进为１８．７４４．５３６．８。工业经济内部结构进一步优化，质量和效益同步提高，规模以上工业企业主营业务收入和利润实现１００４．１亿元和１０３．６亿元，分别增长４３．２％和５６．８％；呼伦贝尔经济技术开发区和岭东工业园区产值分别超百亿元，华能呼伦贝尔能源开发公司产值超百亿元。农牧业产业化带动种养业结构进一步优化，农畜产品加工产业蓬勃兴起，玉米加工产业正在成为经济增长的新亮点。农区牲畜存栏１１７４万头只，占全市牲畜总数的７１％。旅游业已成为拉动增长的重要力量，非公有制经济得到长足发展。

（三）民生社会事业得到显著加强。民生投入达到１１４．８亿元，占地方财政总支出的４５％。千方百计增加城乡居民收入，城镇居民人均可支配收入和农牧民人均纯收入实现１７１４２元和７６４３元，分别增长１５．４％和２１．４％。全面落实新一轮就业组合政策，１２．６万人实现就业，其中高校毕业生就业１．１６万人，城镇登记失业率控制在４．０８％以内。社会保障提标扩面工作扎实推进，城镇职工基本养老、失业、医疗、工伤、生育保险等社会保障标准提高幅度均在１０％以上。城乡居民养老保险参保３８．８万人，城镇企业职工人均养老金达到１４４３元／月。城乡低保保障标准每人每月分别达到２８２元和１５４元。加快社会福利事业发展，爱心家园老年公寓和老年康复养护中心主体工程完工，敬老院集中供养率达到３４％。保障性安居工程开工６２１４３户，完成投资３５．７９亿元，其中森工和农垦分别开工２７５６９户和２６６３户，林区和垦区职工居住条件得到改善。积极推进扶贫开发，当年实现１１万人脱贫，莫旗和鄂伦春旗正式被列为国家级贫困县。认真落实各项惠农惠牧政策，发放农牧民补贴资金２４．１亿元，农牧民人均达到２６２９．６元。落实国家个人所得税调整政策，减轻中低收入者税负５３９０万元。提高了个体工商户营业税和增值税起征点，共减免税收４１０万元。承诺的１０件为民办实事项目全部完成，跨年度的２２项公共公益工程进展顺利。科技创新能力进一步增强，我市首次荣获“全国科技进步先进市”称号。中小学校舍安全工程基本完成，呼伦贝尔学院通过本科教学合格评估。医药卫生体制改革扎实推进，政府办基层医疗卫生机构全部实施基本药物制度，蒙中医服务水平明显提升，基本公共卫生服务能力逐步提高。公共文化服务设施全部免费开放，“五张文化名片”影响力持续扩大。广播、电视人口综合覆盖率分别达到９３．８％和９３．９％。计划生育工作完成控制性指标。统计、供销、体育、质监、人防、气象、工会、地震、残联、档案史志、红十字会、妇女儿童、外事等工作都有新进展。

（四）生态环境和发展条件持续改善。认真落实国家《大小兴安岭林区生态保护与经济转型规划》和草原生态补奖政策，发放草原补奖资金４．８４亿元。全面实施天然林保护二期和退耕还林等工程，重点推进沙区综合治理，治理沙地１０９．２３万亩。公路建设投资６４．２１亿元，总里程突破２万公里，达到２０３３６公里，建成高速公路１６０．１公里，新改建农村公路７７９公里。阿荣旗——扎兰屯铁路建设进展顺利，满洲里——新右旗铁路获自治区批复。呼伦贝尔海拉尔机场改扩建工程竣工，根河通用机场正式通航。水利重点项目开工１０６个，完成投资１０亿元；红花尔基水利枢纽投入使用；实施人畜饮水工程，解决了１０．７８万人和９．４５万头只牲畜的安全饮水问题。电网项目开工８６项，完成投资１５亿元。城市建设项目开工７１４个。中心城新区河东部分建设全面启动，２．２平方公里中心文化区建设进展顺利。扎兰屯、牙克石等地区的新城区建设开始启动。

（五）社会稳定和社会建设成效明显。坚持社会稳定为第一责任，贯彻落实自治区党委、政府关于应对境外敌对势力、维护社会稳定的一系列决策部署，全面开展矿业秩序整顿，关停违规矿山、限期整改不达标企业，打造绿色生态矿山；开展征占用林地、草原和乱开滥垦草原清理整顿等工作，积极排查和化解各种矛盾，有效维护了社会稳定。加大社会治安综合治理力度，群众安全感进一步增强。强化食品药品安全和物价监管，加强安全生产，各类事故死亡人数下降１０．９％，全年没有发生大的森林草原火灾。畅通群众诉求渠道，化解信访积案，妥善解决人民群众的合理诉求，大信访、大调解格局基本形成。推进社区整合，社会管理和服务得到加强。加强政府自身建设，坚持依法行政，自觉接受市人大依法监督和政协民主监督，认真听取各民主党派、工商联和无党派人士意见，办理议案１件、建议１５３件、提案２４０件。加强审计监督和普法教育，行政执法和公正司法水平不断提高。双拥和边防工作进一步加强，呼伦贝尔市和满洲里市再次荣获“全国双拥模范城”称号。精神文明建设成果丰硕，成功承办了全国农村精神文明现场会和全国牧区工作会议。

（六）改革重组取得重大突破。积极整合资源、盘活存量，全力培育新的经济发展主体。整合岭西四个工业园区，组建了呼伦贝尔经济技术开发区。抢抓国家大兴水利机遇，成立了水务投资公司。充实壮大城市投资公司，推进了中心城区基础设施建设。将海拉尔农垦集团和大兴安岭农垦集团进行重组，组建了呼伦贝尔农垦集团公司，为打造农工商一体化企业航母奠定了基础。森工集团按照现代企业制度建立了法人治理结构，为推动林区经济转型发展奠定了体制基础。恢复苏木乡镇建制２７个，基层政权建设得到加强。推进土地草牧场有序流转，全年流转土地３２５万亩、草场９０５万亩。集体林权制度改革主体任务基本完成。积极开展了基层医疗卫生机构综合改革。事业单位和文化体制改革有序推进。

（七）深入研究落实国家政策。抢抓国家支持地方发展政策密集出台的良好机遇，努力把政策优势转化为产业优势和经济优势。针对国家出台的《关于进一步促进内蒙古经济社会又好又快发展的若干意见》、《大小兴安岭林区生态保护和经济转型规划》、草原生态保护补助奖励机制、《关于加快水利改革发展的决定》等政策，深入研究，分解梳理出了１７大类２６０个具体项目，组建项目班子，制定节点规划，实施专项推进。抢抓政策机遇的共识已经形成，政策效应将联动显现。

各位代表，过去的一年，成绩来之不易。这是自治区党委、政府和市委正确领导的结果，是全市各族人民团结奋斗的结果，是市人大、政协有效监督和大力支持的结果，也是社会各界关心和帮助的结果。在此，我代表市人民政府，向全市各族人民，向为呼伦贝尔建设和发展作出贡献的同志们，向所有关心和支持呼伦贝尔的朋友们，表示衷心的感谢和崇高的敬意

在看到成绩的同时，也要清醒地认识发展中存在的矛盾和差距：经济总量小，企业规模小；投资不平衡，地区差距拉大；项目储备不足，发展后劲不强。经济结构不优，农牧业基础地位还不巩固，工业主导地位尚未形成，服务业水平有待提升，非资源型产业和战略性新兴产业培育需要有新的突破，经济结构调整任务艰巨。城乡基础设施薄弱，制约发展的瓶颈依然存在，经济社会发展的保障能力不强。民营经济发展不足，县域经济活力不强，缺少发展的内生动力。农牧林产业组织化程度低，产业链条短，产品初级化、低端化特征明显，产业化程度不高。生态保护与建设任务繁重，节能减排压力巨大。民生支出需求刚性增长，收支矛盾突出，就业和再就业压力较大。部分干部工作漂浮，忧患意识、危机意识不强，发展环境需要优化，行政效能有待提高。一些影响发展和稳定的深层次问题和矛盾仍然存在。对此，我们务必高度重视，切实加以解决。

二、２０１２年主要任务

２０１２年是贯彻落实市第三次党代会精神的第一年，也是深入实施“十二五”规划承上启下的一年。做好今年的工作，保持经济社会又好又快发展，任务繁重，压力巨大。一方面，世界经济增速放缓，欧债危机继续蔓延，贸易保护主义抬头；国内经济下行趋势明显，实体经济经营困难，节能减排与生态约束加剧，保持经济快速增长压力加大。另一方面，要特别看到，我们也面临着诸多有利条件和一系列政策叠加机遇，我市经济形态多元、市场相对稳定、出口依存度低、国有大中型企业处于主导地位，这些条件和优势，有利于我们更好地应对国际国内经济环境变化。只要我们全面落实《关于进一步促进内蒙古经济社会又好又快发展的若干意见》等政策，加大投融资力度，积极承接发达地区产业转移，大力培育非资源型产业和战略性新兴产业，切实加大本土企业培育力度，全面加强同俄蒙和东北地区经济合作，走好点状发展、集约发展、和谐发展之路，妥善处理经济发展与环境保护的关系，我们完全可以继续保持经济社会快速发展的强劲势头。

今年政府工作的总体要求是：全面贯彻落实自治区第九次党代会和市第三次党代会精神，以“美丽发展、科学崛起、共享繁荣”为统领，以做大经济总量为核心，以投资拉动、项目建设、结构调整为重点，以改革开放为动力，大力推进新型工业化、城市化、农牧业产业化进程，大力改善生态环境和基础设施条件，大力发展民生和社会事业，大力加强文化建设、社会建设、民主法制和政府自身建设，大力转变工作作风，以积极进取、攻坚克难的信心和行动，全面完成今年各项目标任务。

今年经济社会发展主要预期目标是：地区生产总值增长２０％，固定资产投资增长２５％，地方财政总收入增长２０％，城镇居民人均可支配收入增长１７％，农牧民人均纯收入增长１５％，单位ＧＤＰ能耗控制在自治区要求以内，城镇登记失业率控制在４％以内，人口自然增长率控制在５‰以下，完成自治区下达的节能节水减排任务。

实现上述预期目标，今年重点抓好七个方面的工作：

（一）以投资拉动、项目建设和结构调整为重点，做大工业经济总量。全年完成工业投资４７０亿元，实施市本级工业重点项目１００个，规模以上工业增加值增长２３％以上。

一要继续壮大传统优势特色产业。紧紧抓住煤电、新型煤化工、有色金属和建材等资源型产业，加强运行调度，积极协调解决好企业生产中的用电、用水、融资、运输等问题，确保现有企业按市场需求，发挥最大产能。煤炭行业要重点推进大唐谢尔塔拉７００万吨露天矿等１０个项目，当年投资４０亿元，产能达到９２００万吨。电力行业要重点推进华电莫旗２×３５万千瓦热电联产、华能牙克石２×２０万千瓦热电联产等１５个项目，当年投资６３亿元，电力装机达到８２１万千瓦。煤化工行业要重点推进华电２×６００万吨褐煤精炼多联产等１２个项目，当年投资１１０亿元，形成２０万吨甲醇、６８万吨合成氨和１１０万吨尿素生产能力。有色金属行业要重点抓好中国黄金１０万吨铜冶炼和驰宏２０万吨铅锌冶炼等１６个项目，当年投资３８亿元，形成日处理８万吨矿石采选和２０万吨铅锌冶炼能力。建材行业要重点抓好阿荣旗蒙西水泥三期等１０个项目，当年投资１７亿元，形成７００万吨水泥和５００万吨水泥熟料生产能力。

二要在新的产业培育上有新突破。围绕现有优势产业搞配套，延伸产业链条，形成产业集群。着力推进农畜产品加工产业，务求取得新突破，重点抓好玉米转化、肉类加工等１２个项目着力推进机械加工制造业，重点抓好山东能源工程机械等６个项目，当年投资２９亿元；着力发展生物制药产业，重点抓好海拉尔鸿洋药业二期工程等３个项目，当年投资５０亿元以上。

三要狠抓“双百亿工程”，培育骨干企业和重点园区。争取呼伦贝尔经济技术开发区升级为国家级园区，建设莫旗工业发展基地，各旗市区要采取切实措施，全力抓好工业园区建设，真正实现点上开发、面上保护。重点抓好园区基础设施建设，加大招商力度，创新融资方式，完善土地储备，增强园区自我发展能力。积极探索创新园区管理体制，着力在园区发展规划、产业定位、核心项目引进上下工夫，延长产业链，发展循环经济。

四要加大扶持力度，推动小微企业发展。要把培育扶持小微企业作为增强地方经济活力和增加就业的重要载体，鼓励引导企业通过技术改造和科技创新增强自我发展能力。积极落实国家和自治区扶持小微企业发展政策，努力争取上级发展基金，同步配套完善各级财税支持政策，建立健全信贷考核制度，多渠道融资，推动小微企业发展。

（二）突出抓好农牧业产业化，加快农牧林区经济发展。加强农牧业科技创新，加大水利等农牧林区基础设施建设力度，提高农牧业综合生产能力。推动粮食总产量稳定增长，优化畜群结构，加快林区经济转型，促进农牧林区经济繁荣。

一要强化政策引导，壮大龙头企业。在尽快做大呼伦贝尔农垦集团的同时，着力培育同联、阜丰、雀巢等产业化骨干企业，继续大力引进新的大型企业集团，做大做强一批产业化龙头企业。

二要加强基地建设，调整种养结构。继续推进耕地、草牧场有序流转，将土地向大型龙头企业和专业合作组织集中，提高集约化水平。围绕玉米、乳、肉等优势产业，加大政策资金扶持力度，下大力气推动种植业和养殖业基地建设，全年玉米种植面积要达到７００万亩，肉羊出栏要达到７００万只以上。

三要抓住政策机遇，发展节水农业。抓住国家大兴水利的难得机会，充分发挥我市水资源丰富的优势，努力发展高效节水农业，新增节水灌溉面积５０万亩。组织实施２０万亩国家“节水增粮”等一批农田水利基础设施项目。

四要加强基础设施建设，建设新农牧林区。推进农牧林区水利、电网、交通、通讯等基础设施建设，投资６亿元，新增通村油路和农牧林场油路６４５公里；投资６亿元，实施农村电网改造工程。继续实施人畜饮水安全工程，抓好农村危旧房改造、环境整治等工作，进一步改善农牧林区面貌。

（三）加快旅游和物流业发展，提高现代服务业发展水平。积极推动旅游、物流、文化、金融等产业发展，为全市经济社会发展提供新的支撑和保障。加快旅游业发展。整合旅游资源，搭建旅游投融资平台，引入大型旅游企业，市场化开发建设和经营运作景区。继续抓好呼伦贝尔民族文化园、蒙兀室韦民族文化园、白鹿岛和红花尔基等景区建设。加快星级酒店建设，提高旅游接待能力。加大冬季旅游开发和营销力度，集中打造海拉尔——满洲里——阿尔山黄金旅游线路，启动森林火车游、森林小镇和林区家庭游项目，办好冰雪旅游节，使森林游和冰雪游成为旅游的新亮点。加大农区旅游产品开发力度，逐步实现农牧林区旅游同步推进、四季旅游均衡发展。举办好第三届那达慕大会。深入开展旅游环境综合治理，切实规范旅游经营服务行为。旅游接待人数增长１８％以上，旅游业总收入增长２０％。加快现代物流业发展。全面推进满洲里口岸国际物流带、滨洲干线物流带和岭东、岭上、岭西物流园区建设，打造新的经济增长点。重点支持海拉尔、满洲里、牙克石、扎兰屯等地区打造区域性物流中心，全力抓好海拉尔中俄蒙国际物流园、牙克石综合物流产业园、扎兰屯岭东商贸物流园等项目建设。努力提升商贸服务业。加快城乡商业网点规划建设，积极发展各类专业市场，打造新型综合商业体，开工建设红星美凯龙等大型专业市场。深入推进连锁经营、电子商务和“万村千乡市场工程”建设，大力开发社区商业、物业、家政等服务业。全面推动金融、房地产、文化产业、商务会展、交通运输、邮电通讯、研发咨询、供销仓储等现代服务业加快发展。要进一步加大扶持力度。在投资、财税、价格、土地等方面制定更具有针对性的政策和措施，促进服务业快速发展，尽快成长为新的支柱产业。

（四）强力推进生态保护和基础设施建设，提升经济社会发展的保障能力。要一以贯之推进生态保护与建设。以生态市建设为统领，在统筹抓好天保二期、草畜平衡、补奖政策落实等各项生态建设工程的同时，重点抓好沙区综合治理，今年要继续坚持任务和投资“两不减”的要求，高质量完成１００万亩沙区综合治理任务。加大环境执法力度，严惩破坏生态环境行为。继续开展绿色、生态、环保、和谐矿山建设活动。要坚持不懈推进城市化进程。理顺呼伦贝尔新城区管理体制，完善城市建设投融资平台，明确城市发展建设主体，要在房屋土地征收、基础设施建设、企事业单位入驻、社会服务配套等方面实现新突破。继续推进海拉尔火车站改造、“三馆一湖”和天骄大桥等重点工程建设。加大市区共建力度，加快推进中心城区供水、供热等基础设施建设和街道改造。有序推动扎兰屯和牙克石等旗市新区的规划和建设。努力完善城市功能，投资５３亿元，完成道路、供水、供热、燃气等２６３个基础设施项目建设。切实提高供热保障能力，积极应对极端天气。完成中心城区供水管网改造工程，保证广大居民饮水安全。力争完成房屋建筑面积１０００万平方米，投资达到１５０亿元。确保完成《呼伦贝尔城市总体规划》修编，加快各旗市区所在地控制性详细规划编制，规划和建设必须突出特色，形成林区、农区、牧区、口岸地区特色鲜明的城市风格。要大力加强基础设施建设。公路投资７０亿元，建设里程１３８２公里，其中高速公路２９２公里。开工建设中心城区东出口路，启动南出口和城市环路前期工作，加快推进海拉尔客运总站和扎兰屯等客运站建设。铁路投资２７亿元，实现阿荣旗——扎兰屯铁路全线贯通，开工建设满洲里—新右旗铁路和工业园区铁路专用线，积极做好海拉尔——黑山头、阿荣旗——莫旗铁路的前期工作。民航以打造呼伦贝尔通用航空机场群和１小时时空圈为目标，进一步完善根河通用机场试点工作，确保鄂伦春机场交付使用，加快呼伦贝尔海拉尔机场迁扩建项目论证，做好扎兰屯支线机场和新右旗、莫旗通用机场前期工作。继续巩固并积极开辟境内外旅游航线。水利要争取扎敦水利枢纽工程开工建设，确保扬旗山水库年底蓄水。抓好晓奇水利枢纽、尼尔基水库下游等三大灌区、格尼河等十九个中型灌区项目的前期工作。电网投资２６亿元，建设８３项输变电工程。确保扎兰屯５００千伏变电站开工建设，加强市内电网建设，改变目前市域内局部窝电、整体缺电的状况。

（五）深化改革开放、扩大区域合作，增强发展的动力与活力。要不断创新和完善科学发展的体制机制，努力构建多层次、宽领域、全方位的对内对外开放格局。

一要持续推进重点领域改革。深化农村牧区改革，有序加快土地和草牧场流转，发展多种形式的适度规模经营。深化集体林权制度改革，支持森工、农垦、地方林业等国有企业改革，规范国有资产收益征缴和国有资产运营行为。大力推进水利、旅游、林业和文化领域资源整合。深入推进事业单位绩效工资制度改革和医药卫生体制改革。完善市与各旗市区财政体制，实施以结构性减税为重点的积极财政政策，进一步提高财政保障和政府宏观调控能力。

二要全面扩大对内对外开放。加强与周边地区的合作，努力实现交通对接、产业对接、资源互补、旅游互动。利用境内外两种资源和两个市场，最大限度地拓展发展空间。积极主动融入东北老工业基地，加强与东北三省的产业、项目和基础设施对接。全面推动满洲里重点开发开放试验区建设，积极推进中蒙跨境合作园区前期工作，加快沿边开发开放步伐。

三要着力开展招商引资，承接产业转移。要围绕产业规划和产业定位，突出优势产业，切实提高招商引资的针对性和有效性，在承接产业转移、引进项目和资金上取得新进展。在项目的承接和引进上要做到：凡是不利于生态环境保护的项目不上，凡是影响群众利益的项目不上，凡是不符合国家产业政策、低水平重复建设的项目不上。

四要大力发展非公有制经济。用足用活政策，优化发展环境，加大金融扶持力度，积极鼓励和引导非公企业围绕优势特色产业搞延伸，围绕重点项目搞协作，围绕大型基地搞配套。全市非公有制经济占ＧＤＰ比重保持在５０％以上。

（六）以改善民生为根本，加快各项社会事业发展步伐。要继续加大投入，切实保障和改善民生，提高公共服务均等化水平，让全市各族人民共享改革发展成果。

一要千方百计促进增收。要健全完善企业职工工资正常增长机制，落实国家提高营业税起征点政策，减轻个体工商户和小微企业税费负担，积极落实各项惠民政策，多渠道增加城乡居民收入。

二要千方百计扩大就业。要大力发展服务业和劳动密集型产业，鼓励和扶持自主创业，以创业促进就业，努力实现社会就业更加充分。要统筹解决好农牧林区富余劳动力、下岗失业人员、退役军人等重点人群的就业问题，继续把高校毕业生就业放在首位，确保９９００名高校毕业生实现就业。

三要切实提高社会保障水平。进一步调整优化公共财政支出结构，优先保障民生。完善基本养老、基本医疗等社会保障制度，将“老工伤”人员全部纳入工伤保险统筹管理。实现城乡居民社会养老保险全覆盖，在机关和事业单位开展生育保险。提高城乡居民最低生活保障标准，加大对低收入群体帮扶救助力度。努力保持物价总体稳定。

四要全力加大扶贫开发力度。把扶贫作为头号民生工程，以加快贫困地区发展为根本，以增加贫困人口收入为核心，以较少民族和国贫、区贫县为重点，全面落实国家和自治区扶贫政策，统筹资源，加强协作，积极推进各项扶贫开发工程，努力构建专项扶贫、行业扶贫和社会扶贫“三位一体”的大扶贫格局，确保完成３万贫困人口脱贫的目标。

五要加大保障性住房建设力度。继续实施保障性安居工程，积极引导牧民进入城镇生活。开工建设保障性住房４６０５１套、２３７万平方米投资３５亿元，尽快使城乡困难群众的居住条件得到改善。六要大力发展社会事业。加快民族文化大市建设，积极推进艺术馆新馆建设，确保市图书馆新馆竣工并对外开放。大力发展文化产业，打造文化精品，促进文化繁荣发展。以建设创新型呼伦贝尔为目标，加快建设区域技术创新体系，努力提高自主创新能力。持续加大教育投入，加快推进城乡教育一体化，促进各级各类教育均衡发展，全面实现高中阶段免费教育。加强基层医疗卫生体系建设，大力扶持发展蒙中医药事业，推进基本公共卫生服务均等化。严格食品药品安全监管，切实加大专项整治力度，确保人民群众饮食用药安全。广泛开展全民健身运动，不断提高体育竞技水平。强化人才开发利用，积极推进人力资源市场建设。全面完成广播电视无线覆盖工程。切实加强人口与计划生育工作，稳定低生育水平，提高出生人口素质。重视发展公益慈善事业，积极做好残疾人和老龄工作。落实民族宗教政策。统筹抓好统计、审计、气象、质监、档案史志和外事等工作。

今年继续为群众办十件实事，一是实施呼伦贝尔医改示范区工程，推进城乡医疗服务均等化。二是实施农村牧区安全饮水工程，解决１３万人安全饮水问题。三是实施牧民进城工程，切实改善牧民生活条件。四是为全市牧民免费赠阅蒙文版《呼伦贝尔日报》，丰富牧民精神文化生活。五是成立呼伦贝尔食品安全检测中心，保障人民群众饮食安全。六是建立气象灾害预警发布系统，增强应对气象灾害的能力。七是实施促进就业工程，新安排城镇就业５万人。八是为高龄老人发放高龄补贴，提高老年人生活质量。九是提高城镇居民医疗保险标准，惠及５２万人。十是为全市城镇低保家庭发放一次性生活补贴。

（七）全面排查和化解社会矛盾，维护社会和谐稳定。始终坚持稳定是第一责任，确保民族和谐、社会稳定、边疆安宁。

一要为“十八大”召开营造良好氛围。今年我们党要召开第十八次全国代表大会，各级政府要切实增强政治意识、大局意识、责任意识，集中精力，细化责任，扎实工作，确保不给国家和自治区添乱。

二要大力化解社会矛盾。推进矛盾纠纷排查化解工作制度化、常态化，定期开展矛盾纠纷排查，强化地方主要领导责任，及时采取有效措施化解社会矛盾。畅通信访渠道，完善信访工作机制，努力把矛盾化解在基层、化解在源头。

三要推进平安呼伦贝尔建设。加强社会治安综合治理，严密防范和严厉打击各类违法犯罪活动，加强公共安全宣传教育和应急演练，建立健全灾害事故和公共安全事件预警、应急处置体系，增强应急指挥协调能力。加强党管武装工作，抓好国防动员，着力推进军警民边防体系建设。继续深入开展双拥模范城创建活动。认真落实安全生产责任制，确保人民群众生命财产安全。

四要创新社会管理。深入推进嘎查（村）级事务民主化管理，提高群众自我服务和发展能力。加强社区文体活动场所建设，提高社会服务管理水平，全市所有社区办公活动场所面积要达到３００平方米以上。建设苏木乡镇周转房，改善基层干部工作和生活环境，完成第七届嘎查（村）换届选举工作。

五要加强民主法制建设。认真执行人大及其常委会的各项决议、决定，自觉接受监督。积极支持政协履行政治协商、民主监督、参政议政的职能。认真办理人大代表议案、建议和政协委员提案，主动听取各民主党派、工商联、无党派人士及各人民团体的意见和建议。支持工青妇等群团组织开展工作。推进“六五”普法，加快法治呼伦贝尔建设进程。广泛开展群众性精神文明创建活动，筹办好撤盟设市十周年庆祝活动。

三、加强政府自身建设

市委决定，今年在党政机关中开展“加强机关纪律作风建设活动”和“万名干部转变作风、提高素质、集中培训教育活动”，政府要以此为契机，全面加强自身建设，着力打造为民、高效、廉洁的政府。

（一）强化宗旨观念，打造为民政府。各级政府是人民的政府，为人民群众谋福祉是政府全部工作的出发点和落脚点。一要端正对人民群众的态度。要突出人民群众的主体地位，作决策、办事情都要广泛征求人民群众的意见，坚持做到问政于民、问计于民，虚心向人民学习，真心维护群众利益。二要密切同人民群众的关系。要带着对人民群众的深厚感情深入基层、深入群众，及时了解和掌握群众最关心、最迫切、最现实的生产生活问题，把人民群众的需要作为各级政府施政的第一信号，密切政府与群众的鱼水关系。三要真心实意为人民群众排忧解难。要把群众利益放在首位，只要是群众关注的事情，就竭尽全力去抓；只要是群众期盼的事情，就义不容辞去办；只要是涉及群众利益的问题，就想方设法去解决。千方百计解决好群众就业、就学、就医、住房和柴米油盐问题，切实做到对人民负责、受人民监督、为人民造福、让人民满意。

（二）提高执行能力，打造高效政府。一要提高抓发展的能力。投资和项目是呼伦贝尔可持续发展的核心和关键。当前和今后相当长时期内，全市的经济发展主要还是靠投资拉动。我们必须深入研究、开阔视野、扎实工作，着力提高抓项目抓投资的能力和水平，以大投资和大建设推动呼伦贝尔实现大发展。二要善于抢抓机遇。面对当前中央支持内蒙古又好又快发展、新一轮西部大开发、大小兴安岭生态保护与经济转型、水利、农业科技等多重政策机遇，我们必须主动应对，深入研究，认真谋划，攻坚克难，通过抢抓机遇促进地区发展。三要坚持解放思想。在招商引资、资源配置等事关经济社会发展的关键环节，要敢于解放思想，打破常规，谋长远、算大账，讲信誉、求双赢，切实提供优惠政策和优质服务，坚持不懈地打造良好发展环境。四要确保政令畅通。政府各工作部门要谋大事、顾全局、管本行，在全市发展大局中找方位、定坐标，不折不扣地贯彻市委、政府的决策部署。

（三）突出制度建设，打造廉洁政府。以强化行政审批中心建设为重点，继续深化行政审批制度改革，进一步清理和规范行政许可项目及非行政许可的行政审批事项，精简办事程序。要坚持依法行政，加强政风建设，深入推进民主评议行风政风工作，加大政务公开力度，建立健全群众对部门和行业作风建设的监督机制，严格规范和监督行政执法行为，推动权力在阳光下运行。要强化对重大决策、重大资源配置、重大项目安排以及大额度资金使用的监督，重点对工程项目招投标、土地出让招拍挂等领域进行监督检查，坚决查处房屋征地拆迁、违规占用草场和乱开滥垦草原等损害群众切身利益的突出问题。加强领导干部廉洁自律，认真解决不同程度存在的形式主义、官僚主义、消极懈怠和奢侈腐败等问题。切实强化行政监察和审计监督，严格责任追究和行政问责，全面提高廉政建设水平。努力建设人民满意的政府。

各位代表，今年经济社会发展的目标任务已经明确。让我们在自治区党委、政府和市委的正确领导下，团结带领全市各族人民，解放思想坚定信心，开拓进取，扎实工作，不断开创我市改革开放和现代化建设的新局面，以优异的成绩迎接党的十八大胜利召开！
